# Spatial and temporal patterns of the emerging tick-borne pathogen *Borrelia miyamotoi* in blacklegged ticks (*Ixodes scapularis*) in New York

**DOI:** 10.1186/s13071-020-04569-2

**Published:** 2021-01-14

**Authors:** F. Keesing, D. J. McHenry, M. H. Hersh, R. S. Ostfeld

**Affiliations:** 1 Program in Biology, Bard College, Annandale-on-Hudson, NY 12504 USA; 2 Alson H. Smith Jr. Agricultural Research and Extension Center, Virginia Tech, Winchester, VA 22602 USA; 3 Department of Biology, Sarah Lawrence College, Bronxville, NY 10708 USA; 4 Cary Institute of Ecosystem Studies, PO Box AB, Millbrook, NY 12545 USA

**Keywords:** *Borrelia miyamotoi*, Blacklegged tick, Emerging infectious disease, *Ixodes scapularis*, Reservoir host, Disease ecology

## Abstract

*Borrelia miyamotoi*, a bacterium that causes relapsing fever, is found in ixodid ticks throughout the northern hemisphere. The first cases of human infection with *B. miyamotoi* were identified in 2011. In the eastern USA, blacklegged ticks (*Ixodes scapularis*) become infected by feeding on an infected vertebrate host, or through transovarial transmission. We surveyed *B. miyamotoi* prevalence in ticks within forested habitats in Dutchess County, New York, and identified possible reservoir hosts. To assess spatial variation in infection, we collected questing nymphal ticks at > 150 sites. To assess temporal variation in infection, we collected questing nymphs for 8 years at a single study site. We collected questing larval ticks from nine plots to estimate the amount of transovarial transmission. To evaluate potential reservoir hosts, we captured 14 species of mammal and bird hosts naturally infested with larval blacklegged ticks and held these hosts in the laboratory until ticks fed to repletion and molted to nymphs. We determined infection for all ticks using quantitative polymerase chain reaction. The overall infection prevalence of questing nymphal ticks across all sites was ~ 1%, but prevalence at individual sites was as high as 9.1%. We detected no significant increase in infection through time. Only 0.4% of questing larval ticks were infected. Ticks having fed as larvae from short-tailed shrews, red squirrels, and opossums tended to have higher infection prevalence than did ticks having fed on other hosts. Further studies of the role of hosts in transmission are warranted. The locally high prevalence of *B. miyamotoi* in the New York/New England landscape suggests the importance of vigilance by health practitioners and the public.
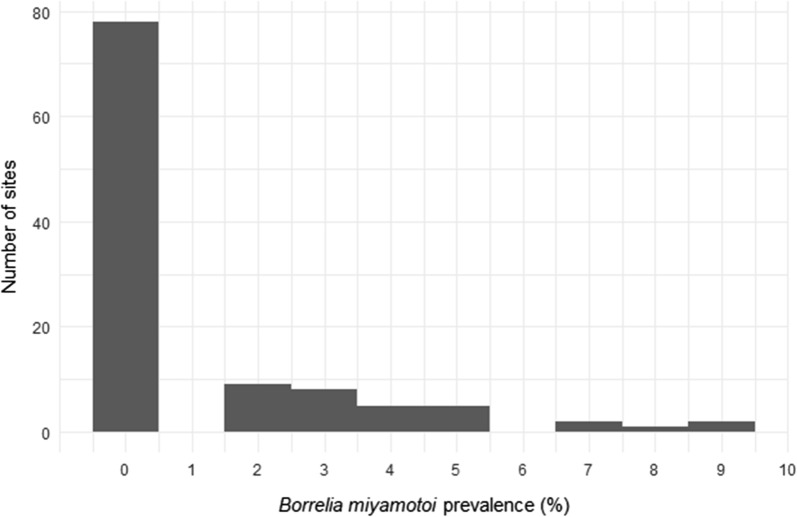

## Introduction

First described from ticks in Japan [9], *Borrelia miyamotoi* is a member of a group of bacteria that cause relapsing fever. Since its discovery in Asia, *B. miyamotoi* has been found in North America [21, 26] and Europe [8]. A congener of *Borrelia burgdorferi*, the pathogen that causes Lyme disease, *Borrelia miyamotoi* has been identified in all of the major tick vectors that transmit the Lyme bacterium among hosts, including *Ixodes scapularis* in the eastern USA [26], *Ixodes pacificus* in the western USA [20], and *Ixodes persulcatus* and *Ixodes ricinus* in Eurasia [8, 9]. It has also been found in vectors that specialize on non-human hosts, including *Ixodes dentatus*, which feeds primarily on birds and rabbits [11].

Although *B. miyamotoi* had been found in ticks from across the northern hemisphere, its role as a human pathogen was recognized only in 2011, when 46 patients in Russia tested positive for *B. miyamotoi* infection [24]. Infected patients suffered high fevers and other influenza-like symptoms [24]. Relapsing fever occurred in 11% of these patients and 5% developed a bulls-eye rash (erythema migrans) [24]. In 2013, the first patients in the USA were identified, with cases in New Jersey and Massachusetts [10, 17]. The role of *B. miyamotoi* in human cases of tick-borne illness is now being more broadly investigated (e.g. 7, 32).

In North America, *B. miyamotoi* occurs at low (< 5%) prevalence in ticks at the sites where it has been identified [1, 6, 16, 19–21, 26, 29, 30, 34]. Ticks can acquire *B. miyamotoi* infection in three ways. First, based on laboratory studies, *B. miyamotoi* can be transmitted transovarially from infected female ticks to their larval offspring [3, 12, 25, 26]. Second, ticks can acquire infection through co-feeding, in which infected ticks transmit infection directly to uninfected ticks feeding on the same host at the same time [26]. Finally, ticks can acquire infection by feeding on hosts infected with *B. miyamotoi*, though the efficiency of transmission appears to be lower for *B. miyamotoi* than it is for *B. burgdorferi* [26]. Once ticks acquire infection, they appear to maintain infection through adulthood [26].

Limited data are available on which host species infect feeding ticks. In Connecticut, nymphal ticks infected with *B. miyamotoi* were collected from an eastern chipmunk (*Tamias striatus*), three raccoons (*Procyon lotor*), an eastern gray squirrel (*Sciurus carolinensis*), and three pine voles (*Microtus pinetorum*) [1, 13]. In Michigan, northern cardinals (*Cardinalis cardinalis*) were responsible for feeding over 70% of the *I. dentatus* ticks that were removed from birds and later found to be infected with *B. miyamotoi* [11], suggesting that cardinals were more likely to infect ticks than other bird species in the study. However, ticks in the study were removed before feeding to repletion, which might have affected patterns of transmission. No ticks collected from eastern cottontails (*Sylvilagus floridanus*) were infected with *B. miyamotoi* [11]. In Tennessee, wild turkeys (*Meleagris gallopavo*) had high seroprevalence of antibodies against *B. miyamotoi* [27]. Studies on other common wildlife species in endemic areas are lacking.

In the northeastern USA, *Borrelia miyamotoi* circulates in the blacklegged tick (*I. scapularis*), the same tick vector that harbors a suite of other pathogens of humans, including *Borrelia burgdorferi*, as well as *Anaplasma phagocytophilum* and *Babesia microti*, which cause anaplasmosis and babesiosis, respectively. In previous studies, co-infections of ticks with *B. miyamotoi* and *B. burgdorferi* have been found to be neither more nor less frequent than expected by chance [1], but see Tokarz et al. [29].

Recent research in parts of Europe and the USA has shown that tick infection prevalence is highly variable at a large geographic scale [5]. For example, infection prevalence at individual sites in 13 counties in California varied from a high of 15.4% of ticks infected to a low of 1.2%, with more than half of sites having no infected ticks.

To address the potentially emerging public health challenge posed by *B. miyamotoi* transmission and consequent disease, we analyzed patterns of infection in ticks in Dutchess County, New York, an area of high incidence of several tick-borne diseases [29]. In a recent survey, infection prevalence for *B. miyamotoi* in Dutchess County was reported as quite low, with 0.5% of ticks infected at the one site sampled [5]. To determine spatial variation in tick infection with *B. miyamotoi* at a regional scale, we collected questing nymphal ticks from >150 sites in Dutchess County over 2 years. We also collected questing larval ticks to determine the frequency of infection resulting from transovarial transmission. Using ticks previously collected for studies of other pathogens, we conducted a preliminary investigation on a suite of vertebrate hosts to determine which species might infect blacklegged ticks with *B. miyamotoi*.

## Methods

### Collecting ticks from forests

We sampled questing nymphal ticks (*I. scapularis*) in June 2011 (107 sites) and June 2012 (53 sites) in forested locations throughout Dutchess County, New York. We selected sites using a geographic information systems map of forested and non-forested land cover digitized from aerial orthophotos generated in 2009. We generated an initial candidate list of 2500 random points using a random point overlay. These points were then stratified by the percentage of forest cover in the surrounding landscape, to provide equal representation along a gradient of forest cover, from extensively forested to highly fragmented. We eliminated sites when access was poor or property owners could not be located or recruited. Details on site selection and sampling locations are provided in Hersh et al. [15].

Between 2006 and 2013, we also collected questing nymphal ticks from six 2.25-ha plots at a single study site at the Cary Institute of Ecosystem Studies in Millbrook, New York. Forests at the Cary Institute are typical of the eastern deciduous forests of New York and New England. The site is dominated by oaks (*Quercus rubra* and *Quercus prinus*) in the overstory, with primarily oak and sugar maple (*Acer saccharum*) seedlings, maple-leaved viburnum (*Viburnum acerifolium*), witch hazel (*Hamamelis virginiana*), and ironwood (*Ostrya virginiana*) in the understory. More information about the Cary Institute study sites and tick sampling protocols are provided in Ostfeld et al. [23].

At all sites at which we collected questing nymphal ticks, we did so by dragging corduroy cloths (1 m^2^) along 400-m transects in each site once or twice in a given year during the annual peak in nymphal questing activity [22]. We collected ticks from the cloths every 15-30 m and froze questing nymphs in liquid nitrogen upon collection. Larvae were flash-frozen in liquid nitrogen in pools of ten. To estimate prevalence at each site, we tested 20-30 nymphs individually (i.e. not in pools).

To test for transovarial transmission, we collected questing larval ticks in August 2013 from nine plots distributed throughout the 1000-ha grounds of the Cary Institute using the same techniques described above. We tested pools of ten larvae. Larvae for these pools were randomly selected from multiple drag samples within each plot to avoid dominance by specific areas within plots.

### Collecting ticks from hosts

To determine which species play a role in infecting larval ticks with *B. miyamotoi*, we determined whether ticks were infected following larval meals on seven mammal and two bird species. For detailed methods, see Hersh et al. [14]. Briefly, we captured host individuals in central Dutchess County, New York, during the peak abundance of larval blacklegged ticks (*I. scapularis*), from July to September in 2008, 2009, and 2010. We held captured individuals for 3 days in cages with wire mesh floors. Cages were suspended over pans lined with wet paper towels so that ticks could be collected after feeding to repletion and dropping from hosts. We maintained engorged larvae in moistened glass vials until they molted into the nymphal stage and then froze them in liquid nitrogen and stored them at -80 °C. We conducted all animal care and husbandry with approval from the Institutional Animal Care and Use Committee of the Cary Institute of Ecosystem Studies. We analyzed tick infection for individual hosts that produced at least 15 larvae that molted into nymphs. Host species for which we had adequate samples of ticks included short-tailed shrews (*Blarina brevicauda*), veeries (*Catharus fuscescens*), Virginia opossums (*Didelphis virginiana*)*,* gray catbirds (*Dumetella carolinensis*), southern flying squirrels (*Glaucomys volans*)*,* wood thrushes (*Hylocichla mustelina*), striped skunks (*Mephitis mephitis*), white-footed mice (*Peromyscus leucopus*), raccoons (*Procyon lotor*)*,* gray squirrels (*Sciurus carolinensis*)*,* masked shrews (*Sorex cinereus*), eastern chipmunks (*Tamias striatus*), American red squirrels (*Tamiasciurus hudsonicus*), and American robins (*Turdus migratorius*).

### Extracting and amplifying DNA

We extracted total genomic DNA from ticks using either the DNeasy or DNeasy 96 Blood and Tissue kit (Qiagen, Hilden, Germany) or the Gentra PureGene Tissue Kit (Qiagen). *B. miyamotoi* was detected using quantitative polymerase chain reaction (qPCR) as described by Platonov et al. [24]. Briefly, we used a TaqMan Probe with 5’-6FAM and 3’-TAMRA (Life Technologies, Grand Island, NY) and iQ Supermix (Bio-Rad, Hercules, CA). Thermal cycling was conducted in a C1000 Thermal Cycler with the CFX96 Real-Time System (Bio-Rad). Ultrapure water served as negative controls. The qPCR target sequence was amplified from SCID+ *B. miyamotoi*-positive mouse blood, provided by Sam Telford III (Tufts University, North Grafton, MA) and cloned into the pCR2.1-TOPO plasmid (Life Technologies). Plasmid extracted from confirmed clones was used as our positive control. Three replicates per tick were amplified. A DNA was considered positive for *B*. *miyamotoi* if at least one replicate was called positive as determined by the default settings for the qPCR machine software (CFX Manager Software 3.0; Bio-Rad).

### Analysis

Larval infection from pooled samples was calculated with the Pooled Prevalence Calculator ([28], http://epitools.ausvet.com.au) using maximum likelihood estimation. To determine an overall estimate of prevalence, we calculated prevalence independently for each of the nine plots from which we collected larvae, and then took the mean of those values.

## Results

### Spatial variation

**Fig. 1:**
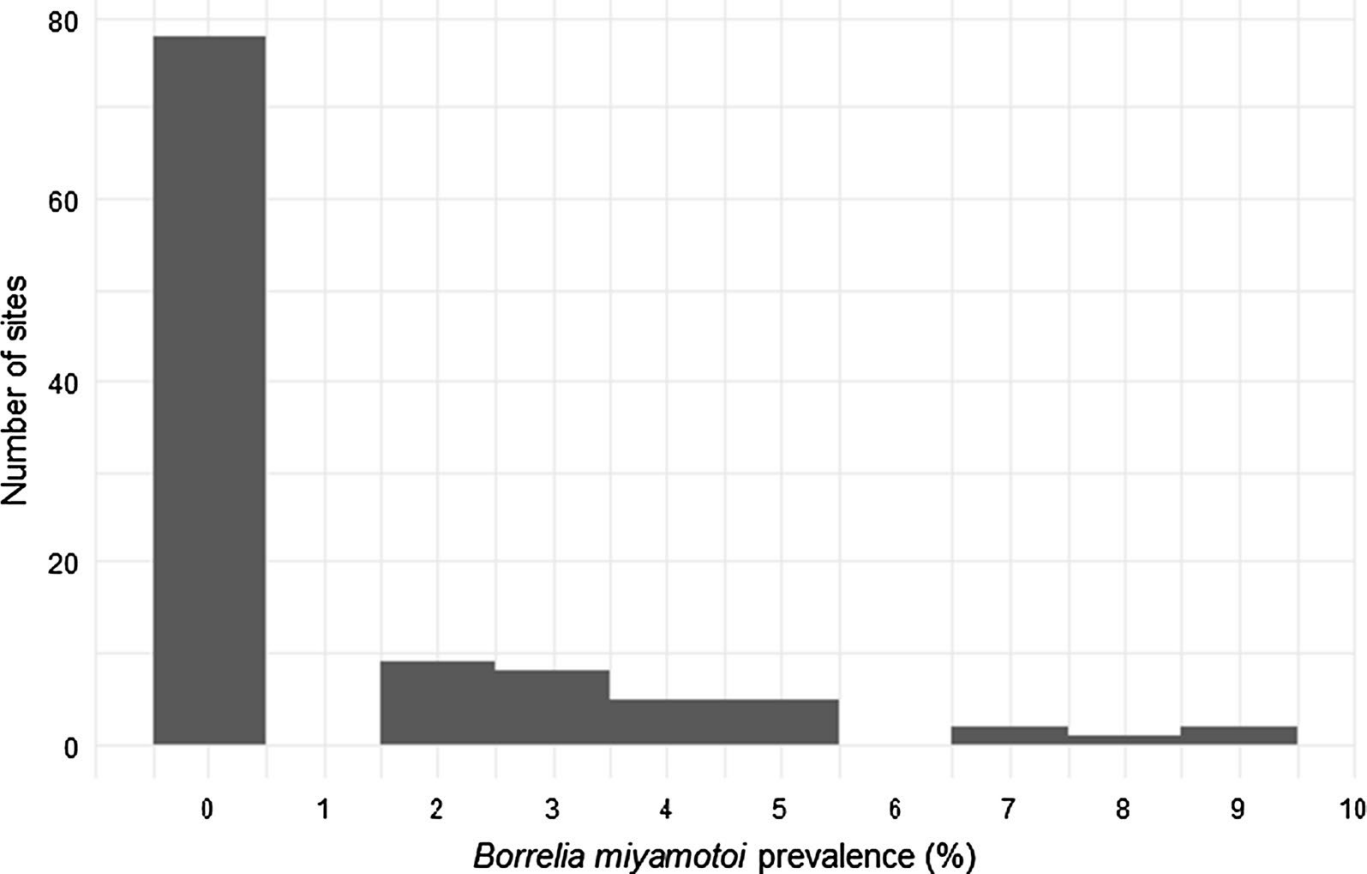
Prevalence of infection with *Borrelia miyamotoi* in nymphal ticks from Dutchess County, New York, collected at 107 sites in 2011 and 53 sites in 2012. Data from the 51 sites sampled in both years are pooled. At each site, prevalence was estimated from a sample of 20-30 ticks

For our regional sampling, we collected a total of 3647 nymphal ticks at 107 sites in 2011 and 53 sites in 2012. Fifty-one of these sites were sampled in both years, while 58 sites were sampled in only 1 year. The mean infection prevalence of ticks summed over both years was 1.16% (2.09% SD). Across both years, the maximum prevalence we detected at an individual site was 9.1% (Fig. 1; Additional file 1: Table S1). At 70.6% of sites (77 out of 109 total), we detected no *B. miyamotoi* (Fig. 1; Additional file 1: Table S1). For the 51 sites we sampled in both years, there was no strong correlation in infection prevalence across years (Kendall’s τ = 0.154).

### Temporal variation

**Fig. 2:**
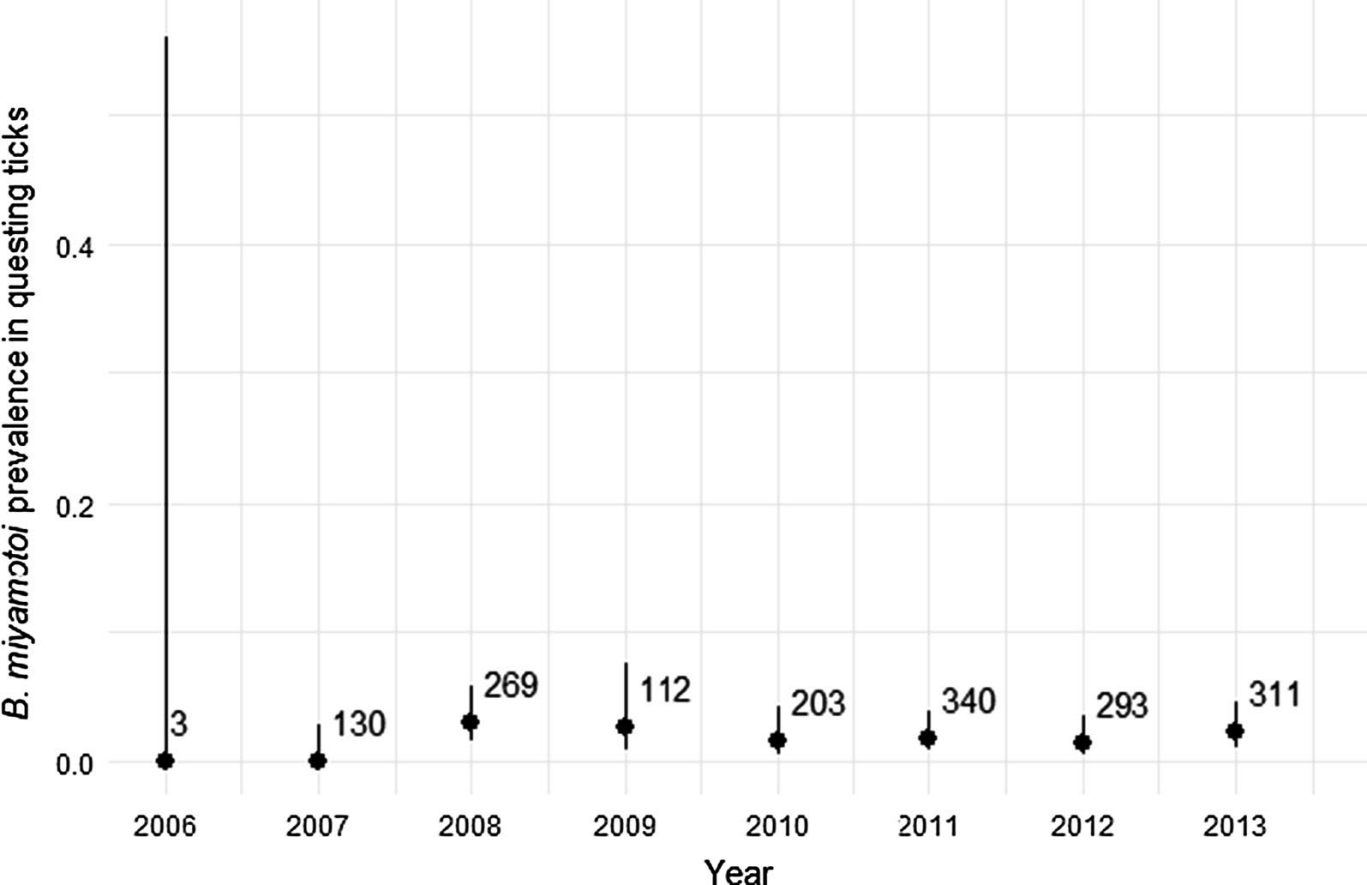
Proportion of ticks infected with *B. miyamotoi* at a single site from 2006 to 2013.* Bars* represent 95% confidence intervals; *numbers* on figure indicate sample sizes

At the single site we sampled over 8 years, there was no significant temporal trend (Fig. 2). Although we collected no ticks infected with *B. miyamotoi* in 2006 and 2007, the 95% confidence intervals for these years overlap with those for subsequent years. After detection of the first infected nymphs in 2008, we found that 1–3% of questing nymphal ticks were infected at this site each year with no apparent increase or decrease through time.

### Larval infection

To determine levels of transovarial transmission in the field, we tested 216 pools of ten larvae from nine plots, with 23-26 pools from each plot. Eight of the 216 pools tested positive for *B. miyamotoi* (0.4% ± 0.2% SE). The mean infection prevalence of larvae, calculated from the pooled data for each of the nine plots, was 0.4%, or four infected larvae per 1000 larvae. Four of the nine plots had infected larvae, and infection prevalences at these plots varied from a low of 0.4% to a high of 1.8%. The remaining five plots had no infected larval pools.

### Reservoir assays

We determined infection with *B. miyamotoi* for 3280 ticks that had fed on 181 individual hosts of 14 common species of vertebrate hosts. We tested a mean of 17.3 (± 0.5, SE of the mean) ticks per individual host. Of the ticks we tested, 38 (1.2%) were infected with *B. miyamotoi*. Hosts from which at least one infected tick was obtained were assumed to be infected, allowing us to estimate infection prevalence at the level of host species. Short-tailed shrews (*B. brevicauda*), gray squirrels (*S. carolinensis*), American red squirrels (*Tamiasciurus hudsonicus*), American robins (*Turdus migratorius*), and Virginia opossums (*Didelphis virginiana*) all had prevalence values of 20% or higher, while some hosts, including gray catbirds (*Dumetella carolinensis*) and veeries (*C. fuscescens*), had zero prevalence (Table 1; Additional file 1: Table S1).

**Table 1:** Host species from which replete larval blacklegged ticks were collected, allowed to molt into nymphs, and then tested using quantitative polymerase chain reaction for the presence of *Borrelia miyamotoi*

Species	*n*	Number of hosts infected	Prevalence (%)	Mean ticks tested/host
American robin (*Turdus migratorius*)	20	4	20	17.3
Short-tailed shrew (*Blarina brevicauda*)	29	10	34	18.8
Virginia opossum (*Didelphis virginiana*)	27	6	22	19.7
Southern flying squirrel (*Glaucomys volans*)^a^	7	0	0	12.4
Gray catbird (*Dumetella carolinensis*)	18	0	0	16.6
Striped skunk (*Mephitis mephitis*)^a^	2	0		15.5
White-footed mouse (*Peromyscus leucopus*)	38	2	5	19.7
Raccoon (*Procyon lotor*)	26	2	8	19.3
Gray squirrel (*Sciurus carolinensis*)	20	1	5	17.9
Masked shrew (*Sorex cinereus*)^a^	6	0	0	6.8
American Red squirrel (*Tamiasciurus hudsonicus*)	15	4	2	19.8
Eastern chipmunk (*Tamias striatus*)	23	3	13	16.0
Veery (*Catharus fuscescens*)	22	0	0	20.2
Wood thrush (*Hylocichla mustelina*)	28	1	4	17.7

Because some of the larval ticks might have been infected prior to host feeding (via transovarial transmission), actual transmission from host to tick cannot be specified.

*n* Number of individuals of each host species from which ticks were obtained and tested for infection,* Prevalence* percentage of each host species producing at least one infected tick

^a^Species for which *n* < 15, rendering estimates of prevalence unreliable

## Discussion

At sites in Dutchess County, New York, the overall infection prevalence of questing nymphal ticks with *B. miyamotoi* was just over 1%, though prevalence at individual sites was as high as 9.1%. Larval infection prevalence, and therefore the risk of transmission from bites of larval ticks, was lower, with only 0.4% of questing larvae infected. Short-tailed shrews, red squirrels, and opossums appear to be potential reservoirs for the pathogen, as ticks that had fed on these hosts were highly likely to have acquired infection from these hosts during their larval meal. Our data provide one of the few field estimates of transovarial transmission to larval *Ixodes* ticks [3, 31], and identify a suite of potential new reservoirs for the pathogen.

We did not detect *B. miyamotoi* in nymphal ticks at 70.6% of sites. Our failure to detect *B*. *miyamotoi* at any given site could have been due to sampling error, which is particularly likely with a rare pathogen. If we assume that 1% of ticks are infected with *B. miyamotoi*, there is an 80% chance that we would fail to detect an infection when sampling 20 ticks from a site. Thus, our estimate of the percentage of sites with *B. miyamotoi* is conservative and provides a lower bound of risk of human exposure.

At the 29.4% of sites at which we did detect infection, prevalence was as high as 9.1%, suggesting high spatial variation in risk. Infection prevalence of nymphal *I. scapularis* with *B. burgdorferi* at similar levels has been associated with substantial burdens of Lyme disease in local human populations [18]. Consequently, it is possible that human infections are much more common than has been reported; alternatively, tick-to-human transmission of *B. miyamotoi* might be considerably less likely than tick-to-human transmission of *B. burgdorferi*. The lack of a correlation in prevalence levels between years suggests that local risk may vary substantially from year to year, but only long-term monitoring can establish this definitively. Although our statistical analysis does not support a conclusion of changing prevalence through time, we failed to detect the pathogen during the first 2 years of sampling, which is consistent with its arrival in 2008.

By collecting replete larval blacklegged ticks having fed from known hosts, we provide preliminary information on possible natural reservoirs for *B. miyamotoi* transmission. We found that several species of mammalian and avian hosts, especially American robins, short-tailed shrews, Virginia opossums, and American red squirrels, showed high prevalence of infection, ranging from 20 to 34%. Because some of the host-fed ticks in which we detected *B. miyamotoi* infection might have acquired infection transovarially, and thus were infected prior to feeding on these hosts, we cannot estimate reservoir competence of the vertebrate hosts. Nevertheless, with infection prevalence for questing larval ticks with *B. miyamotoi* at roughly 1%, we suspect that host-to-tick transmission was responsible for the majority of the positive nymphs. Further studies of reservoir status will be required before host species can be compared quantitatively.

*Borrelia miyamotoi* coexists in the northeastern USA with a suite of other tick-borne pathogens including *Borrelia burgdorferi*; *Anaplasma phagocytophilum*, which causes granulocytic anaplasmosis; *Babesia microti*, which causes human babesiosis; and Powassan virus, which causes Powassan viral encephalitis. All of these pathogens initially cause generalized flu-like symptoms in afflicted humans, making differential diagnosis challenging [2, 4]. Blacklegged ticks can be co-infected with > 1 of these pathogens (e.g. [15]), although patterns of co-infection with *B. miyamotoi* have not been thoroughly examined. It is not yet clear whether standard diagnostic tests for Lyme disease and anaplasmosis can differentiate these illnesses from infection with *B. miyamotoi* [2, 24, 33]. Fortunately, evidence to date suggests that *B. miyamotoi* responds to treatment with antibiotics that are commonly used to treat Lyme disease and anaplasmosis [4, 33].

The presence of *B. miyamotoi* in the New York/New England landscape suggests the importance of vigilance by public health practitioners. In principle, the greatest risk for human exposure to *B. miyamotoi* is during May and June in the northeastern USA, coincident with the peak of questing behavior by nymphal ticks. However, in practice, August and September might also prove to be times of risk. Questing larval ticks, which are active during these months, can be infected with *B. miyamotoi*, but health professionals and the public are less familiar with the risk of tick-borne illnesses during late summer.

## Supplementary Information

**Additional file 1: Table S1.**

## Data Availability

Data are available in Additional file 1: Table S1.
